# Workplace Bullying, Compassion Fatigue, and Organizational Silence Among Perioperative Nurses: A Cross‐Sectional Study

**DOI:** 10.1155/jonm/2431898

**Published:** 2026-09-27

**Authors:** Jamshid Eslami, Ali Nasiri, Mohamad Keramati, Amirali Alizadeh

**Affiliations:** ^1^ Department of Operating Room and Anesthesiology, School of Nursing and Midwifery, Shiraz University of Medical Sciences, Shiraz, Iran, sums.ac.ir; ^2^ Research Center for Evidence-Based Health Management, Maragheh University of Medical Sciences, Maragheh, Iran, mrgums.ac.ir

**Keywords:** bullying, compassion fatigue, operating rooms, organizational silence, patient safety, perioperative nursing

## Abstract

**Background:**

In operating room settings, organizational silence can hinder timely communication about safety concerns and may adversely affect surgical care. Drawing on conservation of resources (COR) theory, this study investigated how workplace bullying and compassion fatigue were associated with organizational silence among perioperative nurses. It also examined whether compassion fatigue conditioned the association between workplace bullying and organizational silence.

**Methods:**

A multicenter cross‐sectional study was conducted with 305 perioperative nurses from teaching hospitals in Iran. Participants were recruited through a multistage cluster sampling procedure. Data were obtained using validated measures, including the Workplace Bullying Questionnaire, the compassion fatigue subscale of the Professional Quality of Life Scale, and the Organizational Silence Behavior Scale. Descriptive statistics, Pearson correlation analysis, and hierarchical moderated regression were used to analyze the data.

**Results:**

Perioperative nurses reported moderate scores for workplace bullying and compassion fatigue, while organizational silence scores were comparatively high. Workplace bullying (*β* = 0.35, *p* < 0.001) and compassion fatigue (*β* = 0.27, *p* < 0.001) were each positively associated with organizational silence. The interaction between workplace bullying and compassion fatigue was also positive and statistically significant (*β* = 0.18, *p* = 0.002; total *R* ^2^ = 0.34), indicating that the association between workplace bullying and organizational silence was stronger at higher levels of compassion fatigue. Organizational silence scores were also higher among nurses employed on temporary contracts than among tenured staff.

**Conclusion:**

The findings support a resource‐oriented interpretation within COR theory, suggesting that workplace bullying and compassion fatigue are associated with greater organizational silence among perioperative nurses. Healthcare administrators should consider integrated workplace interventions that combine antibullying policies with psychological support strategies to reduce silence‐promoting conditions and support nurses’ willingness to voice safety‐related concerns.

**Implication for Nursing Management:**

Nurse managers should address organizational silence through integrated strategies that combine antibullying policies, psychologically safe reporting systems, and compassion fatigue–focused support programs. Such interventions may help reduce silence‐promoting conditions and support perioperative nurses’ willingness to raise patient‐safety concerns.

## 1. Introduction

The operating room (OR) represents a high‐reliability clinical environment where complex interdependencies and high‐stakes decisions demand reliable teamwork to prevent serious errors [1]. In such a high‐stakes environment, patient safety relies heavily on effective communication, particularly the ability of team members to “speak up” when they perceive a safety breach or an operational anomaly [2]. Employee voice behavior refers to the discretionary expression of concerns, suggestions, or safety‐related information intended to improve work processes or prevent harm and is, therefore, particularly relevant in perioperative environments where timely communication can affect patient safety [3]. The nursing workforce, often described as the “eyes and ears” of the healthcare system, plays a pivotal role in this surveillance. Perioperative nurses are uniquely positioned to intercept surgical errors, advocate for sterile techniques, and advocate for patients during the vulnerable period of anesthesia [4]. Consequently, a culture that fosters the free flow of information and concerns is an important prerequisite for surgical safety and quality of care.

Despite the critical necessity of open communication, organizational silence remains an important concern in healthcare settings. Organizational silence is defined not as the mere absence of speech but as a collective and often conscious decision by employees to withhold potentially critical information, ideas, or concerns regarding work‐related problems [5, 6]. From the perspective of organizational silence theory, silence is a meaningful employee behavior rather than simply a lack of communication. Employees may remain silent because they believe speaking up will be ineffective, because they fear negative interpersonal or professional consequences, or because they seek to preserve relationships and avoid conflict. In some circumstances, this silence may represent a withdrawal strategy adopted by nurses to navigate complex social dynamics [7]. The magnitude of this issue is well‐documented; historically, the joint commission has identified communication failures as the root cause of over 70% of sentinel events in hospitals [8]. Previous evidence also suggests that fewer than 10% of healthcare professionals actively confront a colleague when safety protocols are violated [9, 10]. In the high‐stakes context of the OR, organizational silence may reduce opportunities for early error detection, timely correction of unsafe practices, and collective problem‐solving [11]. From a nursing management perspective, these mechanisms suggest that reducing organizational silence requires not only encouraging individual nurses to speak up but also creating psychologically safe reporting systems, addressing bullying behaviors, and strengthening nonpunitive leadership responses [2, 12, 13].

In exploring the antecedents of this silence, researchers have identified various barriers, yet increasing attention is being directed toward adverse interpersonal dynamics of the OR, specifically “workplace bullying.” In hierarchical settings, bullying is not merely an interpersonal conflict but a reflection of power imbalances that can create an intimidating atmosphere [14, 15]. In the highly pressurized environment of the OR, such mistreatment is frequently operationalized in the literature as “disruptive behavior,” “incivility,” or “lateral violence.” Empirical evidence indicates that perioperative nurses may be particularly exposed to these behaviors, with the prevalence of disruptive behaviors and lateral violence frequently exceeding 60%—and reaching up to 85%—in some surgical settings [16]. Theoretically, workplace bullying may contribute to organizational silence by weakening psychological safety and increasing the perceived interpersonal cost of speaking up. Under these conditions, nurses may become less willing to express concerns or challenge unsafe practices, particularly when they anticipate retaliation, humiliation, or lack of managerial support [12, 13]. However, the extent to which this dynamic prevails across different cultural contexts remains a subject for further empirical investigation.

Beyond these external dynamics, the pressures predisposing nurses to silence are not solely external. Perioperative nurses confront a distinct internal challenge known as “compassion fatigue” (CF). Often described as the “cost of caring,” CF originates from continuous exposure to patient trauma and suffering, distinguishing it from general job burnout [17, 18]. Nurses suffering from CF experience symptoms such as emotional numbness and severe depletion of psychological energy [19]. Research logic suggests that “speaking up” is an extra‐role behavior requiring substantial cognitive and emotional effort. Therefore, it is plausible that nurses afflicted with CF may resort to silence not necessarily out of fear, but due to a lack of sufficient energy resources to engage in workplace challenges [20, 21]. Nevertheless, the precise role of this internal resource‐related strain in shaping nurses’ willingness to speak up, particularly in comparison to external stressors, has received limited empirical scrutiny.

The convergence of these external and internal factors can be coherently explicated through the lens of the conservation of resources (COR) theory Hobfoll [22]. In the context of the present study, workplace bullying may be understood as an external psychosocial stressor that threatens interpersonal safety and professional self‐worth, whereas compassion fatigue may reflect an internal state of emotional and psychological resource depletion. When these conditions co‐occur, COR theory suggests that nurses may be more likely to conserve their remaining resources by avoiding high‐cost interpersonal behaviors, including voicing concerns or speaking up [3, 23]. Thus, organizational silence is conceptualized here as a possible resource‐conservation response in a resource‐depleting work environment, rather than as direct evidence of resource loss itself.

Despite these theoretical considerations, the existing literature still leaves an important gap. Previous studies have often examined workplace bullying, burnout‐related constructs, or organizational silence as separate phenomena; however, less is known about how workplace bullying and compassion fatigue are jointly and interactively associated with organizational silence among perioperative nurses. This issue is especially relevant in the Iranian hospital context, where recent empirical evidence has identified communication and organizational challenges related to speaking up, including nurses’ silence about patient safety, barriers to voicing clinical concerns, limited involvement in decision‐making, and workplace conditions associated with safety‐related silence [7, 24, 25].

To address this gap, the present study examined the associations of workplace bullying and compassion fatigue with organizational silence among perioperative nurses. It also tested whether compassion fatigue moderated the association between workplace bullying and organizational silence. Drawing on COR theory, workplace bullying and compassion fatigue may be understood as stressors that deplete psychological and emotional resources and may increase withdrawal‐oriented responses, including organizational silence. Accordingly, the following hypotheses were proposed:

• Hypothesis 1 (H1): Workplace bullying is positively associated with organizational silence among perioperative nurses.
• Hypothesis 2 (H2): Compassion fatigue is positively associated with organizational silence.
• Hypothesis 3 (H3): Drawing on COR theory, we hypothesized that compassion fatigue moderates the association between workplace bullying and organizational silence, such that this association is stronger among perioperative nurses with higher levels of compassion fatigue.

## 2. Materials and Methods

### 2.1. Study Design and Setting

This study used a descriptive‐correlational, cross‐sectional design and was carried out between March 2026 and April 2026. The research was conducted in teaching hospitals affiliated with two major medical centers in East Azerbaijan province, namely, Tabriz University of Medical Sciences and Maragheh University of Medical Sciences. These hospitals were selected because they represent different surgical care contexts, including specialized trauma services in the metropolitan area of Tabriz and general surgical services in Maragheh. Including hospitals from these two settings allowed the study to capture variation in organizational structure, clinical workload, and perioperative work environments. The manuscript was prepared in accordance with the Strengthening the Reporting of Observational Studies in Epidemiology (STROBE (available here)) recommendations for observational research.

### 2.2. Participants and Sampling

#### 2.2.1. Target Population and Sample Size

The study population consisted of perioperative nurses and surgical technologists employed in the ORs of the selected teaching hospitals. The minimum required sample size was estimated using G ∗ Power software, Version 3.1, based on multiple linear regression analysis. Assuming a medium effect size (*f* ^2^ = 0.15), a significance level of 0.05, statistical power of 0.95, and six predictors, including two main independent variables and four demographic control variables, the initial required sample size was 146 participants.

Because participants were recruited from hospital clusters, the sample size calculation was adjusted to account for the potential similarity of responses among nurses working in the same hospital. Since the intraclass correlation coefficient was not available before data collection, a design effect of 1.95 was applied a priori. This adjustment increased the calculated sample size to approximately 285 participants and helped reduce the possibility of insufficient statistical power resulting from within‐cluster similarity. To compensate for an anticipated 15% rate of nonresponse or incomplete questionnaires, the final recruitment target was set at 330 perioperative nurses. Following data screening and the exclusion of incomplete responses and multivariate outliers, 305 participants remained in the final analytic sample.

#### 2.2.2. Sampling Method

A multistage stratified cluster sampling approach was used to recruit participants from the selected hospitals. This approach was chosen to obtain the required sample size while improving coverage across hospitals with different organizational and geographical characteristics.

In the first stage, the study setting was stratified according to geographical location and university affiliation, resulting in two strata: Tabriz and Maragheh. In the second stage, teaching hospitals with active OR units were identified as hospital clusters. Eight teaching hospitals were included, comprising five hospitals in Tabriz and three hospitals in Maragheh. In the third stage, the number of participants required from each hospital was allocated proportionally to the size of its eligible OR workforce. Therefore, hospitals with larger numbers of eligible perioperative staff contributed more participants to the final sample. Within each hospital, eligible nurses and surgical technologists were selected from verified personnel lists using random numbers.

The final analytic sample included 195 participants from hospitals in Tabriz and 110 participants from hospitals in Maragheh. The numbers recruited from the five hospitals in Tabriz were 53, 44, 38, 42, and 18, respectively. The corresponding numbers from the three hospitals in Maragheh were 50, 30, and 30. Across the eight hospital clusters, the number of included participants ranged from 18 to 53.

### 2.3. Eligibility Criteria

Participants were eligible for inclusion if they (1) had at least an associate degree in nursing or OR technology; (2) had worked continuously in an OR for at least 6 months, ensuring sufficient exposure to the perioperative work environment and its organizational dynamics; and (3) provided written informed consent to participate in the study.

Participants were excluded if they met any of the following conditions: (1) self‐reported active psychiatric disorder or use of medications affecting the central nervous system during the previous 6 months; (2) exposure to a major stressful life event, such as bereavement, divorce, or major trauma, within the previous 3 months; (3) concurrent employment in a nonclinical or exclusively administrative position; or (4) submission of a questionnaire with more than 10% missing responses.

### 2.4. Data Collection Procedure

After obtaining permission from nursing management and OR supervisors in the participating hospitals, data collection was carried out across morning, evening, and night shifts. Collecting data across different shifts was intended to include nurses working under varying clinical conditions and to reduce the likelihood of shift‐related selection bias.

The principal investigator visited the OR units during times that were least likely to interfere with clinical care, such as staff breaks, shift handovers, or scheduled administrative periods. Eligible staff members were approached individually or in small groups, and the researchers explained the purpose of the study, the voluntary nature of participation, and the confidentiality procedures. Participants were informed that choosing to participate or decline would not affect their employment status, professional evaluations, or relationship with hospital managers.

Nurses who agreed to participate received a survey package that included an information sheet, a written informed consent form, and the study questionnaires. Because the questionnaire addressed sensitive issues, including workplace bullying and organizational silence, several procedures were used to protect anonymity and reduce social desirability bias. No identifying information was requested on the questionnaires. After completion, participants placed their questionnaires in plain sealed envelopes and deposited them in a locked collection box located in the OR staff lounge.

Participants were allowed approximately 3–5 days to complete the questionnaires at a convenient time. During follow‐up visits, the research team provided brief reminders and collected the sealed envelopes from the designated box. These procedures were intended to protect participants’ privacy, limit the influence of managers or colleagues on responses, and support more candid reporting of workplace experiences.

### 2.5. Measures

Data were collected using a four‐part survey package consisting of a demographic information form and three standardized questionnaires with established psychometric properties.

#### 2.5.1. Demographic Information Questionnaire

A researcher‐developed demographic form was used to record participants’ background and occupational characteristics. The form included items on age, gender, marital status, educational level, total clinical work experience, and years of experience specifically in the OR.

#### 2.5.2. Workplace Bullying Questionnaire (WPBQ)

Exposure to workplace bullying was measured using the 22‐item WPBQ developed by Einarsen et al. The instrument assesses the frequency of exposure to negative workplace behaviors during the previous 6 months. Items are rated on a 5‐point Likert scale from 1 (*“never”*) to 5 (*“daily”*), with total scores ranging from 22 to 110. Higher scores represent greater exposure to workplace bullying. In the present study, the Persian version validated by Kalantari et al. among Iranian healthcare workers was used. The Persian validation study supported a three‐factor structure, including physically intimidating bullying, person‐related bullying, and work‐related bullying, and reported Cronbach’s alpha values ranging from 0.87 to 0.93 [26, 27].

#### 2.5.3. Professional Quality of Life (ProQOL) Scale

Compassion fatigue was assessed using the ProQOL Scale, which was originally developed by Figley and Stamm and later presented by Stamm in its 30‐item version. The ProQOL evaluates professional quality of life among individuals who provide care to people exposed to trauma or suffering. The scale includes three 10‐item subscales: compassion satisfaction, burnout, and secondary traumatic stress, which is commonly used as an indicator of compassion fatigue. Responses are scored on a 5‐point Likert scale ranging from 1 (*“never”*) to 5 (*“very Ooften”*). Items 1, 4, 15, 17, and 29 are reverse‐scored. For each subscale, scores of 22 or lower indicate low levels, scores of 23–41 indicate moderate levels, and scores of 42 or higher indicate high levels of the corresponding construct [28]. The Persian version was evaluated by Gorji et al., who reported an S‐CVI of 0.91, an intraclass correlation coefficient of 0.96 for the overall instrument, and an internal consistency coefficient of 0.73 [29].

#### 2.5.4. Organizational Silence Behavior Scale (OSBS)

Organizational silence was measured using the OSBS, developed by Yalçın and Baykal for healthcare professionals [30]. This 32‐item scale is rated on a 5‐point Likert scale, ranging from 1 (*“I never remain silent”*) to 5 (*“I always remain silent”*). The instrument consists of four dimensions: silence climate (5 items), fear‐based silence (12 items), silence based on contentment/acquiescence (10 items), and organization‐protection silence (5 items). Higher total scores indicate higher levels of organizational silence. The Persian version translated and validated by Mirzaei et al. was used in this study [31]. Their validation study reported acceptable content validity (CVI > 0.79) and confirmatory factor analysis (CFA) indices supporting construct validity, including *χ* ^2^/df = 2.751 and RMSEA = 0.052. The Persian version also showed high reliability, with an overall Cronbach’s alpha of 0.969 and test–retest stability of ICC = 0.951.

### 2.6. Data Analysis

Data were analyzed using IBM SPSS Statistics, Version 26.0. Before conducting the main analyses, the dataset was checked for missing values and multivariate outliers. Questionnaires with more than 10% missing responses were excluded. Multivariate outliers were detected using Mahalanobis distance criteria and removed, leaving a final analytic sample of 305 participants. The normality of continuous variables was assessed using skewness and kurtosis indices, all of which were within the acceptable range of −2 to +2.

Descriptive statistics, including frequencies, percentages, means, and standard deviations, were used to describe participants’ demographic characteristics and the levels of the main study variables. Internal consistency was assessed using Cronbach’s alpha coefficient. Pearson correlation coefficients were calculated to examine bivariate associations among workplace bullying, compassion fatigue, and organizational silence. Independent‐samples *t*‐tests were used to compare organizational silence scores across categorical demographic variables, including employment status.

A three‐step hierarchical multiple linear regression analysis was performed to test the main study hypotheses and examine the moderating effect. Continuous predictors, including workplace bullying and compassion fatigue, were mean‐centered before creating the interaction term to reduce potential multicollinearity. Regression assumptions were assessed prior to model estimation. Multicollinearity was evaluated using the variance inflation factor (VIF), and independence of residuals was examined using the Durbin–Watson statistic.

For the moderation analysis, the interaction between workplace bullying and compassion fatigue was further tested using the PROCESS macro for SPSS, Model 1. Workplace bullying was specified as the independent variable, organizational silence as the dependent variable, and compassion fatigue as the moderator. Age, gender, OR experience, employment status, and city were entered as covariates. Bias‐corrected 95% confidence intervals were generated using 5000 bootstrap samples. Conditional effects of workplace bullying on organizational silence were examined at low (−1 SD), mean, and high (+1 SD) levels of compassion fatigue. The incremental effect size of the interaction term was estimated using Cohen’s *f* ^2^ based on the change in *R* ^2^.

Common method variance was examined using Harman’s single‐factor test. All measurement items were entered into an unrotated exploratory factor analysis, and the variance explained by the first unrotated factor was inspected to determine whether a single factor accounted for the majority of covariance among the measures.

To evaluate the construct validity of the instruments in the present sample, CFA was conducted using IBM SPSS AMOS, Version 26.0. Model fit was assessed using the chi‐square/degrees of freedom ratio (*χ* ^2^/df), comparative fit index (CFI), Tucker–Lewis Index (TLI), root mean square error of approximation (RMSEA), and standardized root mean square residual (SRMR). Composite reliability (CR) and average variance extracted (AVE) were calculated to examine internal consistency and convergent validity. Discriminant validity was assessed using the Fornell–Larcker criterion and the heterotrait–monotrait ratio of correlations (HTMT).

Because participants were recruited from eight hospital clusters, the potential effect of hospital‐level clustering was considered. Given the relatively small number of clusters, multilevel random‐effects modeling or cluster‐robust standard errors could have produced unstable estimates. Therefore, hierarchical multiple linear regression was used as the primary analytic approach, and a sensitivity analysis was conducted by adding hospital fixed‐effect dummy variables to the final regression model. This analysis was used to determine whether the main effects and interaction effect remained substantively consistent after accounting for between‐hospital differences.

A two‐tailed *p* value of less than 0.05 was considered statistically significant for all analyses.

### 2.7. Ethical Considerations

This study was conducted in strict adherence to the ethical guidelines and principles outlined in the Declaration of Helsinki. The research protocol was formally reviewed and approved by the Regional Ethics Committee of Maragheh University of Medical Sciences (Ethical Code: IR.MARAGHEHPHC.REC.1404.069). Prior to data collection, written informed consent was obtained from all participating nurses. Participants were provided with comprehensive information regarding the study’s objectives, the voluntary nature of their participation, and their absolute right to withdraw at any stage without providing a reason. To prevent any coercion or fear of retaliation—particularly given the highly sensitive nature of exploring workplace bullying and organizational silence—it was explicitly guaranteed that participation would have no bearing on their employment status, performance evaluations, or professional standing. Strict measures were implemented to ensure anonymity and confidentiality. The questionnaires were completely deidentified and coded numerically, containing no names or direct identifiers. Furthermore, the use of sealed envelopes and secure drop‐boxes in the staff lounge ensured that nursing managers or peers could not intercept responses. All resultant electronic datasets were encrypted, stored on a password‐protected drive accessible exclusively to the core research team, and will be securely destroyed after the statutory retention period (typically 5 years postpublication).

## 3. Results

### 3.1. Demographic Characteristics of the Participants

Of the 330 survey packets distributed among perioperative nurses, 316 were returned. After screening for missing data and multivariate outliers, 11 questionnaires were excluded because they had more than 10% missing values or met the criteria for multivariate outliers. The final analytic sample consisted of 305 participants, corresponding to an effective response rate of 92.4%. The mean age of participants was 34.50 ± 7.25 years, with an age range of 23–54 years. The mean length of OR clinical experience was 9.80 ± 6.55 years. Most participants were female (61.0%) and held a bachelor’s degree in nursing or OR technology (89.2%). In terms of employment status, 53.4% of participants were employed under temporary or contractual arrangements. Detailed demographic and occupational characteristics are presented in Table 1.

**TABLE 1:** Demographic and occupational characteristics of the participants (*N* = 305).

Variable	Category	Frequency (*n*)	Percentage (%)
Gender	Male	119	39.0
	Female	186	61.0

Education level	Bachelor’s degree	272	89.2
	Master’s degree or higher	33	10.8

Employment status	Permanent/tenured	142	46.6
	Contractual/temporary	163	53.4

Hospital location	Tabriz	195	63.9
	Maragheh	110	36.1

Work shift	Fixed morning	47	15.4
	Rotating shifts	258	84.6

### 3.2. Descriptive Statistics and Correlation Analysis

Table 2 presents the means, standard deviations, Cronbach’s alpha coefficients, and bivariate correlations for the main study variables. Cronbach’s alpha coefficients ranged from 0.86 to 0.92. The mean score for workplace bullying was 55.20 ± 11.85, and the mean score for compassion fatigue was 32.40 ± 8.12. The mean organizational silence score was 96.75 ± 16.40.

**TABLE 2:** Descriptive statistics, reliability, and Pearson correlation matrix (*N* = 305).

Variables	Mean	SD	Alpha	1	2	3
1. Workplace bullying	55.20	11.85	0.89	1		

2. Compassion fatigue	32.40	8.12	0.86	*r* = 0.34^∗∗^ *p* < 0.001	1	

3. Organizational silence	96.75	16.40	0.92	*r* = 0.46^∗∗^ *p* < 0.001	*r* = 0.41^∗∗^ *p* < 0.001	1

*Note:* Alpha = Cronbach’s alpha coefficient. Statistical test used for correlations: Pearson correlation coefficient.

Abbreviation: SD, standard deviation.

^∗∗^ *p* < 0.01 (2‐tailed).

Pearson correlation analysis showed statistically significant positive correlations among the main study variables. Workplace bullying was positively correlated with compassion fatigue (*r* = 0.34, *p* < 0.01) and organizational silence (*r* = 0.46, *p* < 0.01). Compassion fatigue was also positively correlated with organizational silence (*r* = 0.41, *p* < 0.01).

Harman’s single‐factor test was conducted to assess common method variance. The first unrotated factor explained 34.2% of the total variance, which was below the 50% threshold. Because all variables were assessed using self‐report questionnaires completed by the same respondents at a single time point, common method bias could not be completely excluded.

### 3.3. Measurement Model and Construct Validity

CFA was conducted to examine the construct validity of the study instruments in the current sample. The measurement model produced the following fit indices: *χ* ^2^/df = 2.34, CFI = 0.94, TLI = 0.92, RMSEA = 0.065, and SRMR = 0.052. CR values ranged from 0.87 to 0.93. AVE values ranged from 0.56 to 0.64. For the Fornell–Larcker criterion, the square root of the AVE for each construct was greater than its correlations with other constructs. HTMT values were below 0.85.

### 3.4. Hierarchical Moderated Regression Analysis

A three‐step hierarchical multiple regression analysis was performed to examine the associations of workplace bullying and compassion fatigue with organizational silence and to test the moderating role of compassion fatigue. Workplace bullying and compassion fatigue were mean‐centered before the interaction term was created. VIF values for all predictors ranged from 1.04 to 1.42.

As shown in Table 3, demographic variables, including age, gender, OR experience, employment status, and city, were entered in Step 1. This model explained 7% of the variance in organizational silence (*R* ^2^ = 0.07, *p* < 0.001). In Step 2, workplace bullying and compassion fatigue were added to the model, explaining an additional 23% of the variance (Δ*R* ^2^ = 0.23, *p* < 0.001). Workplace bullying was positively associated with organizational silence (*B* = 0.48, SE = 0.08, 95% CI [0.32, 0.64], *β* = 0.35, *p* < 0.001). Compassion fatigue was also positively associated with organizational silence (*B* = 0.55, SE = 0.11, 95% CI [0.33, 0.77], *β* = 0.27, *p* < 0.001).

**TABLE 3:** Hierarchical regression analysis of factors associated with organizational silence (*N* = 305).

Predictors	B	SE	Beta	*t*	*p* value	VIF
*Step 1: Control variables*						
Age	0.15	0.11	0.08	1.36	0.174	1.18
Gender (female = 1)	3.12	1.15	0.12	2.71	0.007	1.09
OR experience	0.35	0.14	0.16	2.50	0.013	1.22
Employment status (contractual = 1)	5.20	1.25	0.20	4.16	< 0.001	1.14
City (Maragheh = 1)	1.10	1.12	0.04	0.98	0.327	1.04
*R* ^2^ = 0.07, *F* for Step 1 = 4.50, *p* < 0.001						

*Step 2: Main effects*						
Workplace bullying (WB)	0.48	0.08	0.35	6.00	< 0.001	1.31
Compassion fatigue (CF)	0.55	0.10	0.27	5.50	< 0.001	1.28
Delta *R* ^2^ = 0.23, *F* change = 42.15, *p* < 0.001						

*Step 3: Interaction effect*						
WB × CF (interaction)	0.03	0.01	0.18	3.10	0.002	1.42
Total *R* ^2^ = 0.34, delta *R* ^2^ = 0.04, *F* change = 9.61, *p* = 0.002						

*Note:* A sensitivity analysis including seven hospital fixed‐effect dummy variables produced substantively similar results.

In Step 3, the interaction term between workplace bullying and compassion fatigue was added to the model. The interaction term explained an additional 4% of the variance in organizational silence (Δ*R* ^2^ = 0.04, *p* = 0.002). The interaction coefficient was positive and statistically significant (*β* = 0.18, *p* = 0.002). The 95% confidence interval for the interaction term was also statistically significant (*B* = 0.03, SE = 0.010, 95% CI [0.01, 0.05], *p* = 0.002). The incremental effect size for the interaction term was *f* ^2^ = 0.06.

Simple slopes analysis was conducted at low, mean, and high levels of compassion fatigue. At low levels of compassion fatigue (−1 SD), workplace bullying was positively associated with organizational silence (*B* = 0.21, SE = 0.09, 95% CI [0.03, 0.39], *p* = 0.024). At the mean level of compassion fatigue, workplace bullying was positively associated with organizational silence (*B* = 0.45, SE = 0.08, 95% CI [0.29, 0.61], *p* < 0.001). At high levels of compassion fatigue (+1 SD), workplace bullying was positively associated with organizational silence (*B* = 0.69, SE = 0.10, 95% CI [0.49, 0.89], *p* < 0.001).

To examine the robustness of the findings in relation to the hospital‐based clustered sampling structure, a sensitivity analysis was performed by adding seven hospital fixed‐effect dummy variables to the final regression model. The direction and statistical significance of the main associations and the workplace bullying × compassion fatigue interaction term remained substantively unchanged after including hospital fixed effects.

### 3.5. Interaction Plot

A simple slopes plot was generated to illustrate the interaction between workplace bullying and compassion fatigue (Figure 1). Workplace bullying was displayed on the *x*‐axis and organizational silence on the *y*‐axis. Separate slopes were plotted for low (−1 SD) and high (+1 SD) levels of compassion fatigue. As shown in Figure 1, the slope for the association between workplace bullying and organizational silence was steeper at high levels of compassion fatigue than at low levels of compassion fatigue.

**FIGURE 1:**
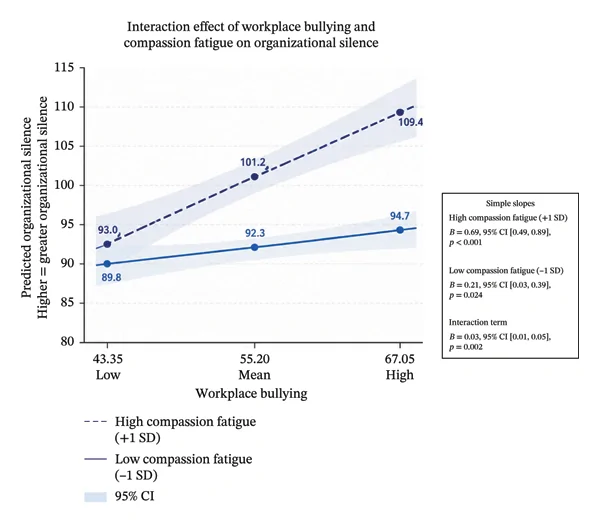
The moderating role of compassion fatigue on the relationship between workplace bullying and organizational silence among perioperative nurses.

## 4. Discussion

The current study aimed to examine the associations of workplace bullying and compassion fatigue with organizational silence among perioperative nurses, while specifically exploring the moderating role of compassion fatigue in this relationship. The findings showed that both workplace bullying and compassion fatigue were positively associated with organizational silence. In addition, compassion fatigue significantly moderated the association between workplace bullying and organizational silence. However, the interaction term explained a statistically significant but relatively modest additional proportion of variance in organizational silence (Δ*R* ^2^ = 0.04). Therefore, the moderation effect should be interpreted as meaningful but limited in magnitude. In other words, the positive relationship between workplace bullying and organizational silence appeared to be stronger among nurses reporting higher levels of compassion fatigue, but the practical magnitude of this interaction should not be overstated. These findings are consistent with a COR‐based theoretical interpretation, suggesting that external psychosocial stressors and internal emotional strain may jointly shape nurses’ willingness to speak up in the OR. However, because resource depletion was not directly measured and the study used a cross‐sectional design, this explanation should be regarded as a theoretical interpretation rather than direct empirical evidence of resource loss.

The moderating role of compassion fatigue was statistically significant; however, its practical magnitude was modest, as the interaction term explained an additional 4% of the variance in organizational silence (Δ*R* ^2^ = 0.04). Therefore, compassion fatigue should not be interpreted as a primary driver of organizational silence. Rather, the findings suggest that compassion fatigue modestly strengthens the positive association between workplace bullying and organizational silence. This interpretation is consistent with COR theory, which proposes that individuals strive to obtain, retain, and protect valued psychological and emotional resources [22]. Workplace bullying may function as an external psychosocial stressor that threatens relational safety and professional self‐worth. At the same time, compassion fatigue may reflect an internal state of emotional strain related to repeated exposure to patient suffering, critical conditions, and surgical emergencies. When these conditions co‐occur, nurses with higher compassion fatigue may have fewer perceived psychological resources available to engage in high‐cost interpersonal behaviors, such as voicing concerns or challenging unsafe practices. From this perspective, organizational silence may be understood as a possible resource‐conservation response, rather than as direct evidence of resource depletion or “resource bankruptcy.” Therefore, healthcare administrators should consider interventions that address both workplace mistreatment and emotional strain among perioperative nurses. These may include antibullying policies, psychologically safe reporting systems, nonpunitive leadership responses, and accessible psychological support programs.

Furthermore, the descriptive and correlational findings of this study offer insights into the psychosocial climate of ORs. The relatively high baseline level of organizational silence, together with moderate levels of workplace bullying and compassion fatigue, suggests that communication‐related barriers may be present in perioperative work environments. The significant positive association between workplace bullying and organizational silence indicates that nurses who reported higher exposure to bullying also reported greater organizational silence. This finding may reflect the role of fear, perceived retaliation, or reduced psychological safety in shaping nurses’ willingness to speak up [32]. Similarly, the significant positive association between compassion fatigue and organizational silence suggests that emotionally strained nurses may be less willing or less able to engage in effortful communication. These findings point to two potentially different pathways to silence: one related to interpersonal threat and another related to emotional strain. However, these pathways should be interpreted cautiously because the cross‐sectional design does not allow causal ordering to be established [33]. Importantly, the significant interaction suggests that when workplace bullying and compassion fatigue co‐occur, the association between bullying and silence becomes stronger, although only to a modest degree. Accordingly, nursing managers should prioritize the prevention of workplace bullying as a central organizational concern, while also addressing compassion fatigue as an aggravating factor that may intensify organizational silence. Recognizing these distinct pathways—fear‐based silence versus exhaustion‐based silence—may help nursing managers tailor organizational communication and psychological support strategies more effectively.

Beyond the primary psychological variables, employment status also emerged as a relevant demographic factor in the baseline regression model. Nurses working under temporary or contractual agreements reported significantly higher organizational silence than their tenured or permanent counterparts. Through the lens of COR theory, job security may be considered a structural resource that supports employees’ willingness to express concerns. Contractual nurses may perceive greater vulnerability when speaking up, particularly if they believe that voicing concerns could affect contract renewal or workplace relationships [34, 35]. In hierarchical and high‐pressure environments such as the OR, this vulnerability may be intensified because speaking up often requires nurses to challenge interpersonal norms, question unsafe practices, or raise concerns about senior colleagues and organizational procedures. For nurses with less secure employment status, these actions may be perceived as professionally risky, even when they are necessary for patient safety. Accordingly, this finding suggests that organizational silence may be shaped not only by interpersonal and emotional factors but also by employment‐related insecurity and perceived organizational power. Contractual employment may reduce nurses’ sense of psychological safety by creating uncertainty about whether their concerns will be received constructively or used against them. This may encourage silence as a protective strategy, particularly in settings where staff perceive limited protection from retaliation, blame, unfavorable evaluations, or non‐renewal of contracts. However, this interpretation should be considered exploratory and requires further longitudinal and qualitative investigation.

The positive association between workplace bullying and organizational silence observed in this study is consistent with a growing body of evidence. Recent studies across diverse cultural contexts, including South Korea Eon Sim and Jang [36] and Egypt El‐Sayed et al. [37], have similarly reported associations between workplace mistreatment, bullying, ostracism, and silence‐related behaviors among nurses. Our findings are also compatible with research from Ghana Wuni et al. [14], which highlighted the adverse implications of bullying for nurses’ work experiences and job performance. Taken together, these studies suggest that workplace bullying may be an important barrier to employee voice in healthcare settings. Nevertheless, the present findings should be interpreted as evidence of association rather than proof that bullying directly causes organizational silence.

A more nuanced interpretation is also needed regarding the motives for silence in perioperative settings. Previous studies in general nursing contexts, such as Zou et al. [5, 38], have suggested that organizational silence may arise from different motives, including prosocial, defensive, or acquiescent reasons. In the present study, the observed associations among bullying, compassion fatigue, and organizational silence may indicate that both interpersonal threat and emotional strain are relevant to silence among perioperative nurses. This interpretation is consistent with studies reporting emotional exhaustion, burnout, and compassion fatigue among OR and surgical nurses in China and Italy [18, 39, 40]. However, because the present study did not directly measure the specific motives for silence, it cannot determine whether the silence reported by participants was predominantly defensive, acquiescent, or prosocial. Future studies should examine these dimensions more directly. Therefore, the present moderation finding should be understood as suggesting that compassion fatigue may intensify the association between bullying and silence, while acknowledging that the additional variance explained by the interaction was relatively modest.

### 4.1. Practical Implications for Nursing Management

The findings of this study offer potentially useful implications for healthcare administrators, OR managers, and policymakers. First, addressing organizational silence may require attention to workplace conditions that enable bullying, incivility, and fear of speaking up. Hospital leadership should move beyond passive awareness and consider consistent implementation of antibullying and antiincivility policies, regardless of the perpetrator’s status or surgical specialty. Establishing secure, anonymous, and independent reporting channels is important so that nurses can voice safety concerns with reduced fear of professional retaliation.

Second, interventions should go beyond merely encouraging nurses to “speak up.” Given the statistically significant but modest moderating role of compassion fatigue, managers should consider strategies that support nurses’ psychological well‐being and reduce emotional strain, such as structured debriefing, resilience training, and fair shift allocation. Furthermore, considering that contractual nurses reported significantly higher organizational silence, hospital administrators should reduce employment‐related vulnerability and foster psychological safety for all staff, regardless of employment status. Practical strategies may include clear nonretaliation policies, confidential reporting channels, inclusive staff meetings, and explicit leadership support for speaking up about patient safety, bullying, workload concerns, or communication failures.

### 4.2. Strengths and Limitations

This study has several strengths. It focuses on the OR, a high‐stakes clinical environment in which communication failures and silence may have important implications for teamwork and patient safety. In addition, the study integrates COR theory to examine the combined relevance of an external psychosocial stressor, workplace bullying, and an internal emotional strain, compassion fatigue, in relation to organizational silence among perioperative nurses. The use of a multicenter sample and validated measurement instruments also strengthens the methodological basis of the study.

However, several limitations should be acknowledged. First, the cross‐sectional design precludes conclusions about causality or temporal ordering among workplace bullying, compassion fatigue, and organizational silence. Therefore, the findings should be interpreted as associations rather than evidence of causal pathways. Although COR theory was used to guide the interpretation of the findings, resource depletion was not directly measured in this study; therefore, COR‐based explanations should be considered theoretical interpretations rather than direct empirical mechanisms. Future longitudinal or prospective studies are needed to examine whether workplace bullying and compassion fatigue precede changes in organizational silence over time.

Second, all data were collected using self‐report questionnaires from the same respondents at a single time point. This may have increased the risk of common method bias and social desirability bias, particularly because workplace bullying, compassion fatigue, and organizational silence are sensitive workplace‐related constructs. Although anonymity, sealed envelopes, and secure drop‐boxes were used to encourage honest responses, common method variance cannot be fully ruled out.

Third, the study used a hospital‐based cluster sampling design. Participants working within the same hospital may have shared similar organizational climates, leadership patterns, staffing conditions, or communication norms. Although the design effect was incorporated during sample size estimation and a sensitivity analysis using hospital fixed effects was performed, residual within‐hospital nonindependence may still have influenced the precision of the regression estimates. Therefore, the standard errors and statistical significance of the associations should be interpreted with caution.

Fourth, residual confounding may remain possible. Although several demographic and occupational variables were controlled in the regression models, other unmeasured factors, such as leadership style, staffing adequacy, workload intensity, psychological safety, team climate, prior exposure to workplace violence, organizational justice, and reporting culture, may have influenced the observed associations.

Fifth, the exclusion criteria may have introduced selection bias. Participants with self‐reported active psychiatric disorders, recent major stressful life events, or recent use of central nervous system–affecting medications were excluded to reduce potential confounding in the assessment of compassion fatigue and related psychological constructs. However, this strategy may have systematically excluded individuals who were more psychologically vulnerable and potentially more likely to report higher levels of workplace bullying, compassion fatigue, or organizational silence. Consequently, the observed levels of compassion fatigue and organizational silence may have been underestimated, and the generalizability of the findings to perioperative nurses experiencing more severe psychological distress may be limited.

Finally, although the sample included perioperative nurses from several teaching hospitals, the findings may not be fully generalizable to private hospitals, nonteaching hospitals, or healthcare systems with different organizational structures and employment conditions. Future studies should include more diverse clinical settings and consider broader inclusion criteria or sensitivity analyses to examine whether these associations differ among nurses with higher psychological vulnerability.

## 5. Conclusion

This study found that workplace bullying and compassion fatigue were positively associated with organizational silence among perioperative nurses. Compassion fatigue also moderated the association between workplace bullying and organizational silence, such that the positive relationship was stronger among nurses reporting higher levels of compassion fatigue. However, because the study used a cross‐sectional design, these findings should be interpreted as associations rather than causal relationships.

From a COR‐based perspective, the findings may suggest that external psychosocial stressors and internal emotional strain jointly shape nurses’ willingness to speak up in the OR. However, resource depletion was not directly measured; therefore, this explanation should be considered a theoretical interpretation rather than direct empirical evidence of a causal mechanism. Nursing managers and healthcare administrators should consider integrated strategies to reduce workplace bullying, strengthen psychologically safe communication systems, and provide accessible psychological support for perioperative nurses.

## Funding

This research did not receive any specific grant from funding agencies in the public, commercial, or not‐for‐profit sectors. All costs associated with data collection and manuscript preparation were personally funded by the authors.

## Disclosure

The study was conducted as part of the authors’ primary employment at Maragheh University of Medical Sciences and Tabriz University of Medical Sciences.

## Conflicts of Interest

The authors declare no conflicts of interest.

## Supporting Information

Additional supporting information can be found online in the Supporting Information section.

## Supporting information


**Supporting Information** STROBE statement—checklist of items that should be included in reports of observational studies.

## Data Availability

The datasets generated and/or analyzed during the current study are not publicly available due to privacy and confidentiality agreements regarding the sensitive nature of the workplace bullying data but are available from the corresponding author on reasonable request.
